# Size matters in non-canonical inflammasome activation and cell-mediated immunity

**DOI:** 10.1016/j.xcrm.2022.100904

**Published:** 2023-01-17

**Authors:** Adam M. Weiss, Aaron P. Esser-Kahn

**Affiliations:** 1 Pritzker School of Molecular Engineering, University of Chicago, 5640 South Ellis Avenue, Chicago, IL 60637, USA

## Abstract

Particulate adjuvants are key components of many approved vaccines, but their mechanism of adjuvanticity is debated. Muñoz-Wolf et al.^1^ find that 50-nm particles maximize cell-mediated immune responses by activating the caspase-11 inflammasome, providing mechanistic insight to particulate adjuvant technologies.

## Main text

Prophylactic vaccines are among the greatest medical advances. Early vaccines were comprised of inactivated pathogens, which intrinsically contained pathogen- and damage-associated molecular patterns (PAMPs and DAMPs). Newer subunit protein and nucleotide-based vaccines can lack these immunostimulatory components and require supplementation with adjuvants.^2^ Particulate adjuvants, including aluminum salts (“alum”), oil-in-water emulsions, and polymer or lipid nanoparticles, have found extensive use in modern vaccines.^2^ These adjuvants are attractive for their low cost, acceptable tolerability, and high efficacy. Furthermore, particulate adjuvants can be engineered to mimic the structure of pathogens and control the spatiotemporal release of vaccine components.^3^ While effective in producing antibody-mediated Th2 immunity, some particulate adjuvants struggle in generating cell-mediated Th1 immunity. Cell-mediated immunity is necessary to destroy damaged cells present during viral infection or cancer.

While several FDA-approved vaccines employ particulate adjuvants, their mode of action remains poorly understood. Particulate adjuvants can stabilize antigen at the injection site and generate inflammation to recruit antigen-presenting cells.^3^ Some argue that particulate adjuvants induce adaptive immune responses independently of inflammation,^4^ while others hypothesize that inflammation generated via activation of inflammasomes is critical for particles’ adjuvant effects. Inflammasomes are damage sensors that become activated by disruptions to homeostasis, such as reactive oxygen species (ROS), and form multi-protein effector complexes.^5^ These complexes activate caspases to initiate secretion of pro-inflammatory IL-1 cytokines, including IL-1α, IL-1β, and IL-18, and inflammatory cell death, termed pyroptosis. IL-1 cytokines are potent mediators of cellular immune responses, though they also cause systemic inflammation if improperly regulated.^5^ Studies have shown that alum can activate the NLRP3 inflammasome to induce IL-1β secretion, but effects of this cytokine secretion on *in vivo* adjuvanticity are unclear.^6^ Adjuvant effects of squalene-based emulsions, meanwhile, were found to be mediated by an IL-1 receptor adaptor protein, MyD88, in an NLRP3-independent fashion.^7^ Finally, lipid nanoparticles were recently shown to induce robust IL-1β secretion *in vivo*.^8^ Variability in the synthesis or physicochemical properties of particulate adjuvants and their *in vivo* immunological readouts has hindered complete mechanistic understanding of these technologies.

Muñoz-Wolf et al.^1^ provide key insights into the role of particle size in immune responses. The authors prepared two classes of polymeric nanoparticles, each varied in diameter from 50 nm to 30 μm. By formulating these particles with a model antigen, ovalbumin, and using them in a prime-boost vaccination model, they found that particle size is critical for cell-mediated immunity. 50- to 60-nm particles, but not larger particles, induced splenic CD8^+^ and Th1-biased CD4^+^ T cell responses, characteristic of cellular immunity. All particle sizes induced antibody-mediated responses. To further characterize the size-dependent cell-mediated response toward 50-nm particles, mice were vaccinated and then implanted with ovalbumin-expressing melanomas. All but one of the mice vaccinated with 50-nm particles and ovalbumin were protected against tumor growth, while fewer than half vaccinated with ovalbumin alone were protected. When CD8^+^ T cells were depleted prior to tumor implantation, protection generated by 50-nm particles was reduced, providing evidence that cell-mediated immunity facilitated tumor regression.

Having generated cell-mediated immunity using 50-nm particulate adjuvants, the authors then sought to identify the mechanism for these adjuvant effects. First, they confirmed a role for IL-1 cytokines using IL-1 and IL-18 receptor knockout (IL-1R^−/−^ and IL-18R^−/−^) mice. Both receptors contributed to the cell-mediated response. When vaccinated with 50-nm particles and ovalbumin, IL-1R^−/−^ mice had deficient Th1-biased CD4^+^ T cell responses, while IL-18R^−/−^ mice had deficient CD8^+^ T cell responses. This “division of labor” has been reported elsewhere^5^ but never confirmed using particulate adjuvants. They initially suspected that IL-1 and IL-18 were produced by the canonical inflammasome effector, caspase-1, but studies using an inhibitor were unsuccessful. They then probed other inducers of IL-1 and IL-18. Caspase-11 forms a non-canonical (i.e., independent of caspase-1) inflammasome.^9^ Several DAMPs activate this sensor, but its role in adaptive immunity is poorly understood.^9^ Using two models, caspase-11 knockout mice or pre-treatment with a pyroptosis inhibitor, the authors provide compelling evidence that caspase-11 inflammasome activation was responsible for cell-mediated immunity induced by 50-nm particles. In both models, CD8^+^ T cell responses in mice vaccinated with 50-nm particles and ovalbumin were reduced to unadjuvanted levels. Finally, with a mechanism identified, the authors characterized how particulate adjuvants activate caspase-11. They found that 50-nm (but not larger) particles induce intracellular ROS production. Moreover, co-administration of 50-nm particles and ovalbumin with a ROS scavenger reduced CD8^+^ T cell responses to unadjuvanted levels. Taken together, these data strongly suggest that 50-nm particulate adjuvants activate caspase-11 inflammasomes via ROS generation to generate cell-mediated immunity.

**Figure 1:**
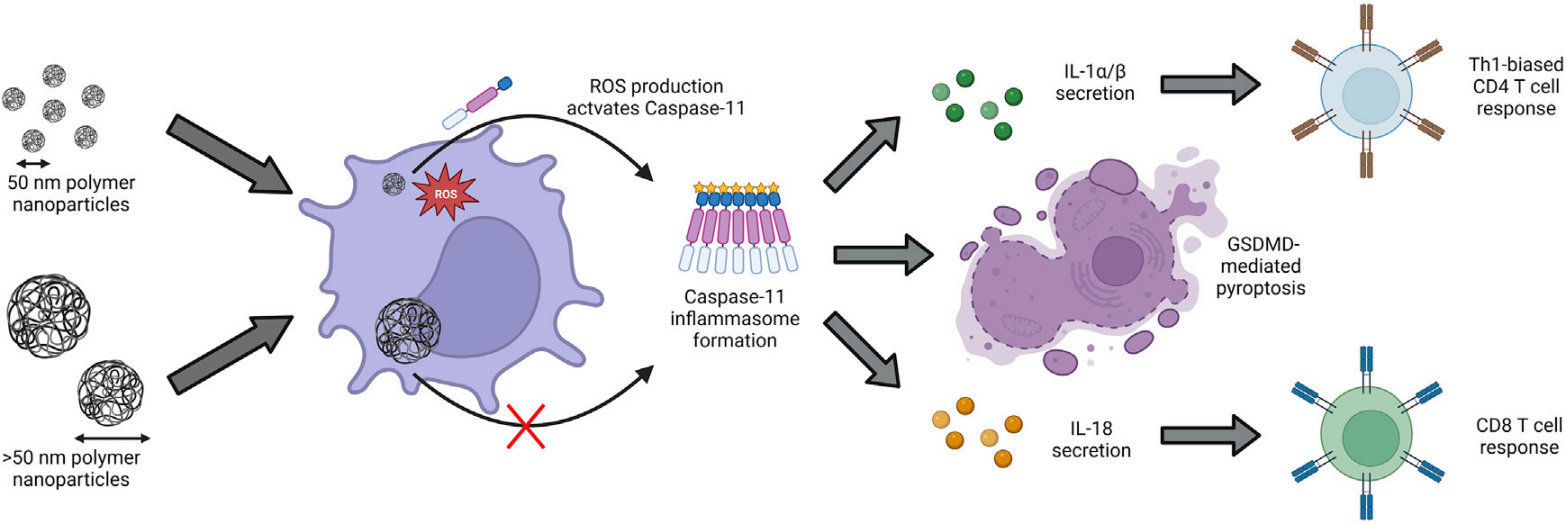
Model of Caspase-11 mediated cellular immunity generated by polymeric nanoparticles 50-nm nanoparticles induced ROS production, while larger particles did not. ROS production facilitated caspase-11 inflammasome activation, resulting in pyroptosis and secretion of IL-1 cytokines. IL-1α/β mediated a Th1-biased, antigen-specific CD4^+^ T cell response, while IL-18 mediated an antigen-specific CD8^+^ T cell response. Created with BioRender.com.

Particle-based adjuvants have gained renewed attention, with lipid- and polymer-based formulations being components of RNA-based vaccines and immunotherapies. While effective, these formulations lack conventional PAMPs, and their mode of adjuvanticity can be unclear.^2^^,^^4^^,^^8^ This work provides evidence that particulate systems activate the caspase-11 inflammasome. Particles comprised of two different polymers induced intracellular ROS production and caspase-11 inflammasome activation, resulting in IL-1 and IL-18 secretion, pyroptosis, and cell-mediated immunity in a size-dependent fashion (Figure 1). 50-nm particles were optimal in facilitating cell-mediated immunity, while larger particles only generated antibody-mediated immunity. It is possible that smaller particles enhance uptake by antigen-presenting cells and/or lymphatic transport to the draining lymph node.^10^ A corollary to this result is that particles >100 nm could be used to prevent unwanted inflammation in contexts such as drug delivery. An open question that remains is whether the role of caspase-11 in cell-mediated immunity can be generalized to other particulate adjuvants and inducers of ROS. While more characterization of this mode of adjuvanticity is still needed, this work provides insight to the role of size, ROS production, and caspase-11 activation in the generation of cell-mediated immune responses by particulate adjuvants and will inform the optimization of new and existing therapeutics.
